# Increased Tau Expression Correlates with Neuronal Maturation in the Developing Human Cerebral Cortex

**DOI:** 10.1523/ENEURO.0058-20.2020

**Published:** 2020-05-28

**Authors:** Kimberly L. Fiock, Martin E. Smalley, John F. Crary, Anca M. Pasca, Marco M. Hefti

**Affiliations:** 1 Department of Pathology, University of Iowa, Iowa City, IA 52242; 2 Department of Pathology, Icahn School of Medicine at Mount Sinai, New York, NY 10029; 3 Department of Neuroscience, Icahn School of Medicine at Mount Sinai, New York, NY; 4 Friedmann Brain Institute, Icahn School of Medicine at Mount Sinai, New York, NY 10029; 5 Department of Pediatrics, Stanford University, Palo Alto, CA 94305; 6 Iowa Neuroscience Institute, University of Iowa, Iowa City, IA 52242; 7 Interdisciplinary Neuroscience Graduate Program, University of Iowa, Iowa City, IA 52242

**Keywords:** Alzheimer’s disease, fetal, neurodegeneration, neurodevelopment, organoid, tau

## Abstract

Although best known for its role in Alzheimer’s disease (AD), tau is expressed throughout brain development, although it remains unclear when and which cell types this expression occurs and how it affects disease states in both fetal and neonatal periods. We thus sought to map tau mRNA and protein expression in the developing human brain at the cellular level using a combination of existing single-cell RNA sequencing (sc-RNAseq) data, RNA *in situ* hybridization (RNAscope), and immunohistochemistry (IHC). Using sc-RNAseq, we found that tau mRNA expression begins in radial glia but increases dramatically as migrating neuronal precursors mature. Specifically, *TBR1*^+^ maturing neurons and *SYN*^+^ mature neurons showed significantly higher mRNA expression than *GFAP*^+^/*NES*^+^ radial glia or *TBR2*^+^ intermediate progenitors. By RNAscope, we found low levels of tau mRNA in subventricular zone (SVZ) radial glia and deep white matter intermediate progenitors, with an increase in more superficially located maturing and mature neurons. By total-tau IHC, the germinal matrix and SVZ showed little protein expression, although both RNAscope and sc-RNAseq showed mRNA, and Western blotting revealed significantly less protein in those areas compared with more mature regions. Induced pluripotent stem cell (iPSC)-derived cortical organoids showed a similar tau expression pattern by sc-RNAseq and RNAscope. Our results indicate that tau increases with neuronal maturation in both the developing fetal brain and iPSC-derived organoids and forms a basis for future research on regulatory mechanisms triggering the onset of tau gene transcription and translation, which may represent potential therapeutic targets for neurodegenerative tauopathies and neurodevelopmental disorders.

## Visual Abstract

**Figure:**
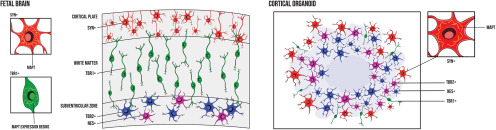

## Significance Statement

Tau is a mediator of neurotoxicity across multiple neurodegenerative diseases, including Alzheimer’s disease (AD) and chronic traumatic encephalopathy (CTE). With the recent failure of β-amyloid-targeted therapies in AD to improve cognitive function, there is increasing interest in tau targeted therapies. Tau is expressed throughout brain development, but the function and normal developmental expression remains unclear. Here, we demonstrate that tau expression begins early during neuronal maturation in both human fetal brain and induced pluripotent stem cell (iPSC)-derived cortical organoids. This work forms the basis for future research into the developmental regulation of tau expression, which may provide future tau-related therapeutic targets.

## Introduction

Tau aggregation and neurotoxicity act as the final common pathway in multiple neurodegenerative diseases, including Alzheimer’s disease (AD), frontotemporal lobar degeneration (FTLD), chronic traumatic encephalopathy (CTE) and primary age-related tauopathy (PART; Morris et al., 2011; Crary et al., 2014). In the case of AD, it is conventionally thought that, although β-amyloid plaques are the first manifestation of disease, tau protein aggregates are a necessary prerequisite for cell death and neurodegeneration (Kametani and Hasegawa, 2018). The recent failure of several β-amyloid reducing therapies in late-stage clinical trial has led to increased interest in tau as a primary therapeutic target in AD and other dementias (Kang et al., 2011; Morris et al., 2011).

Tau is expressed in both rodent and human fetal brain, albeit with developmental changes in both splicing and phosphorylation (Goedert et al., 1989; Jovanov-Milosevic et al., 2012; Hefti et al., 2018). Despite tau knock-out mice showing only subtle neurologic abnormalities (Ikegami et al., 2000; Lei et al., 2012), in vitro studies suggest that tau protein is necessary for normal neurite outgrowth and differentiation (Dawson et al., 2001; Sennvik et al., 2007). Tau also appears to play a role in glutamatergic signaling (Miller et al., 2014; Tracy and Gan, 2017), excitotoxicity (Liang et al., 2009), and epileptogenesis (Liu et al., 2017). Recently, it was demonstrated that human fetal tau has a predominantly short-isoform, hyperphosphorylated phenotype similar to that seen in AD (Hefti et al., 2018, 2019). It remains unclear, however, what cell types express tau in the developing human brain and when in development this occurs. This question is a critical first step in identifying mechanisms regulating tau expression at both the RNA and protein levels which may, in turn, serve as potential targets for tau-reducing therapies.

Induced pluripotent stem cell (iPSC)-derived human brain organoids have become a key tool in developmental neurobiology (Amin and Paşca, 2018; Chuye et al., 2018). Human cortical organoids described by Yoon et al. (2019) and Pasca et al. (2015) are region-specific brain organoids derived through directed differentiation methods to generate dorsal forebrain progenitors, pyramidal cortical neurons, and astrocytes (Pasca et al., 2015; Yoon et al., 2019) Extensive characterization of human cortical organoids through single-cell analyses, transcriptional profiling, and histologic assays demonstrated cytoarchitectural inside-out organization resembling the developing human dorsal forebrain and progressive in vitro differentiation of progenitors into pyramidal neurons of multiple cortical layers and astrocytes (Pasca et al., 2015; Sloan et al., 2017; Yoon et al., 2019). In brief, human cortical organoids contain ventricular zone (VZ)-like and subventricular zone (SVZ)-like proliferative zones rich in neural progenitor cells, including NES^+^ radial glia and TBR2^+^ intermediate progenitors. These proliferative areas are surrounded by cortical plate-like zones rich in pyramidal cortical neurons of multiple subtypes, as well as astrocytes (Pasca et al., 2015; Sloan et al., 2017; Yoon et al., 2019). Importantly, dorsal forebrain human cortical organoids show high reliability across multiple human (h)iPSC lines and have proven valuable for neurodevelopmental disease modeling (Blair et al., 2018; Pasca et al., 2019). Although there is significant interest in using human cortical organoids as model systems in studies of neurodegenerative disease, it is not clear to what degree they recapitulate developmental patterns of tau expression (Ehrlich et al., 2015; Iovino et al., 2015; Silva et al., 2016). We therefore used a combination of single-cell RNA sequencing (sc-RNAseq) data, RNA in situ hybridization (RNAscope), and total tau immunohistochemistry (IHC) to characterize the developmental trajectory of tau expression in human fetal tissue samples and iPSC-derived dorsal forebrain human cortical organoids.

## Materials and Methods

### Tissue samples

Formalin-fixed paraffin-embedded (FFPE) tissue was obtained from the autopsy archives of the Mount Sinai Medical Center and University of Iowa Hospitals and Clinics. Hematoxylin and eosin (H&E)-stained slides cut from each tissue section were reviewed by one of the authors, an experienced developmental neuropathologist, to ensure that they contained cerebral cortex, white matter and SVZ. Layers were defined as described in standard histology references, with white matter including stratified transitional fields 1–6 (also known as the intermediate zone; Ernst et al., 2011). Tissue collection protocols were approved by the Mount Sinai Institutional Review Board (protocol IRB-17-01313). Protocols at the University of Iowa were reviewed by the Institutional Review Board (HawkIRB) and determined to be exempt from review (project 201706772). All methods were conducted in accordance with the relevant guidelines, laws, and regulations. De-identified fresh frozen human fetal brain tissue of either sex was obtained from the NIH NeuroBioBank at the University of Maryland Baltimore. Fixed frozen slides of hiPSC-derived cortical organoids from three cell lines cultured as previously described were provided by Anca M. Pasca (Pasca et al., 2015; Sloan et al., 2018).

### sc-RNAseq data

We analyzed tau (*MAPT*) gene expression data from publicly available sc-RNAseq datasets for fetal brain (Nowakowski et al., 2017) and human dorsal forebrain cortical organoids (Yoon et al., 2019). The former included 48 samples of human fetal cortex ranging from 5.8 to 37 postconceptional weeks (PCW) and the latter included hiPSC-derived cortical organoids derived from three different donors at 105 d of in vitro differentiation. Tissue procurement, cortical organoid differentiation protocol, characterization and quantification of cell types in cortical organoids, and RNA extraction and processing for each dataset are described in detail in the original publications (Nowakowski et al., 2017; Yoon et al., 2019). The genes of interest, including *MAPT*, *TBR1*, *EOMES* (*TBR2*), *NES*, and *SYN*, were extracted from the overall dataset for both fetal and cortical organoid data and organized by lamina (fetal) and cell type (fetal and cortical organoid). Differences in gene expression were assessed using general linear models (glm function in R) using age and either cell type or lamina as covariates. For the purposes of this analysis, cell type was defined using the same markers used for RNAscope (see RNAscope assay methods). All statistical analysis was conducted with R 3.5.3 and RStudio 1.1.

### Code accessibility

All code was written using R 3.5.3 and RStudio 1.1 on a Dell Optiplex 9010 running Windows 10 Enterprise version 1709. All datasets are publicly available through the original cited publication, and code is freely available online at https://github.com/kfiock/tau-maturation.

### RNAscope assay

RNAscope was performed using the RNAscope 3-plex Multiplex Fluorescent v2 Reagent kit (ACDBio, catalog #323100) according to the manufacturer’s directions for FFPE tissue and fixed frozen tissue. Briefly, 5-μm FFPE tissue sections were baked for 1 h at 75°C, deparaffinized using xylene and ethanol, and treated with H2O2 for 10 min at room temperature. Target retrieval was performed using a steamer for 15 min at 85°C, followed by Protease Plus treatment for 30 min at 40°C in a HybEZ Oven (ACDBio). Probes were mixed at a 50:1:1 ratio (C1:C2:C3) and hybridized for 2 h at 40°C in the HybEZ Oven. Slides were stored in 5× SSC overnight. Amplification and detection were performed the following day according to the manufacturer’s instructions.

Fixed frozen cortical organoid sections were rinsed with 1× PBS and baked for 30 min at 75°C, then postfixed with 4% PFA in 1× PBS for 15 min at 4°C. Slides were dehydrated according to manufacturer’s instructions, then pretreated with H2O2 at room temperature for 10 min. Target retrieval was performed as above for 5 min, followed by Protease III treatment for 30 min at 40°C in a HybEZ Oven. Probe application, amplification, and detection were performed as above. Autofluorescence quenching was performed before coverslipping using Vector TrueVIEW kit (Vector Laboratories, catalog #SP-8400) according to manufacturer’s instructors and diluted 1:10.

The following probes from ACDBio were used: Hs-EOMES C1 (catalog #429691), Hs-MAPT C2 (catalog #408991-C2), Hs-SYP C3 (catalog #311421-C3), Hs-TBR1 C1 (catalog #425571), and Hs-NES C1 (catalog #486341). TSA Plus fluorophores (PerkinElmer) were reconstituted according to the manufacturer’s instructions and diluted in the provided TSA buffer at the following concentrations: fluorescein (NEL741E001KT) 1:750 for FFPE tissue and 1:400 for fixed frozen tissue, cyanine 3 (NEL744E001KT) 1:1500 for FFPE tissue and 1:400 for fixed frozen tissue, and cyanine 5 (NEL745E001KT) 1:1000 for FFPE tissue and 1:400 for fixed frozen tissue. Slides were visualized using a Leica SP8 CSU confocal microscope with Leica Las-X software and an Olympus VS120 6-Slide slide scanner with Olympus OlyVIA software. Images for fetal tissue were taken using a 63× objective and zoomed 121% on the Las-X software, then the brightness was adjusted using the same settings in Adobe Photoshop. Images for cortical organoids were taken using a 63× objective, then the brightness was adjusted using the same settings in Adobe Photoshop.

### RNAscope probe validation

The *MAPT* RNAscope probe was validated using the RNAscope 2.5 HD Manual Assay kit (catalog #322430, ACDBio) on FFPE brain tissue from mice injected with a human MAPT P301L construct as a positive control and wild-type mice as a negative control. Mouse tissue sections were the kind gift of Dr. Gloria Lee and were processed as above for RNAscope on FFPE tissue following the manufacturer’s guidelines for the 2.5 HD Manual Assay kit.

### Quantification

Quantification of gene expression on RNAscope-stained tissue sections was done blinded to region and sample by one of the authors under the supervision of an experienced developmental neuropathologist. Two images of each of three regions for five cases total (fetal) and two images of the cortical plate-like region, remote from any rosettes, of each of five cortical organoids (from three cell lines) at each age were taken using a 63× oil-immersion objective on a Leica SP8 confocal microscope All images were taken using identical settings. Channels were then split and thresholded using Fiji (ImageJ) with default settings. For each image, the percent area of the total area positive for *MAPT* was calculated, then averaged across technical replicates. Neuronal maturation markers (*TBR1*, *TBR2*, *SYN*) were done to show co-expression with tau and were thus not quantified.

### Immunohistochemistry

FFPE tissue blocks were cut at 4–5 μm in thickness in the usual fashion. Sections were placed on charged slides and baked overnight at 70°C. IHC was performed on a Ventana Benchmark XT according to manufacturer’s directions. Antigen retrieval was done using CC1 (citric acid buffer) for 1 h followed by primary antibody incubation with an anti-total tau antibody (HT7, catalog #MN1000, Thermo Fisher Scientific, RRID: AB_2314654) for ∼30 min. Slides were visualized using an Olympus BX40 brightfield microscope with an Olympus DP27 camera and CellSens software.

### Western blotting

Manual dissection of germinal matrix and SVZ, white matter, and cortical plate on frozen tissue sections was performed by an experienced neuropathologist. Sections of each area were homogenized in a high salt buffer containing 650 mm Tris-HCl, 2 mm EDTA, 750 mm NaCl, 500 mm NaF and Halt protease, and phosphatase inhibitor cocktail (catalog #78440, Thermo Fisher Scientific) using Fisherbrand Pre-Filled Bead Mill Tubes (catalog #15-340-153, Fisher Scientific) in a Fisherbrand Bead Mill 4 Homogenizer (catalog #15-340-164, Fisher Scientific) for 20 s at 5 m/s. For each area of each sample, 30 μg of protein was run on a 10% Mini-PROTEAN TGX Stain-Free Precast gel (catalog #4568036, Bio-Rad), blotted to PVDF membrane, and stained with the same anti-tau antibody used for IHC as above at 1:5000 dilution. Horseradish peroxidase-labeled secondary anti-mouse antibody (catalog #PI-2000, Vector Laboratories, RRID: AB_2336177) was used at 1:5000 dilution and detected by Clarity Western ECL substrate (catalog #1705060, Bio-Rad). Chemiluminescence was measured using a ChemiDoc Touch Imaging System (catalog #1708371, Bio-Rad). Recombinant tau (catalog #SP-495, Boston Biochem) was used as a positive control and bovine serum albumin (BSA; RPI) was used as a negative control. Quantification of Western blottings was done using the gel analysis feature on Fiji (ImageJ) to measure the area under the curve for each band, which was then normalized to the loading control.

### Statistical analysis

sc-RNAseq data were analyzed using general linear models (glm function in R) and confidence intervals (confint function in R) using R 3.5.3 and RStudio 1.1. Boxplots were created in R using the accessible code as mentioned above. Cumming estimation plots were created to express mean difference of each region from quantification above and a two-sided permutation t test was used to calculate p value (Ho et al., 2019). The effect sizes and confidence intervals (CI) are reported as following: effect size [CI width lower bound, upper bound].

## Results

### Tau mRNA expression increases with neuronal maturation by sc-RNAseq in human fetal tissue

**Figure 1.**
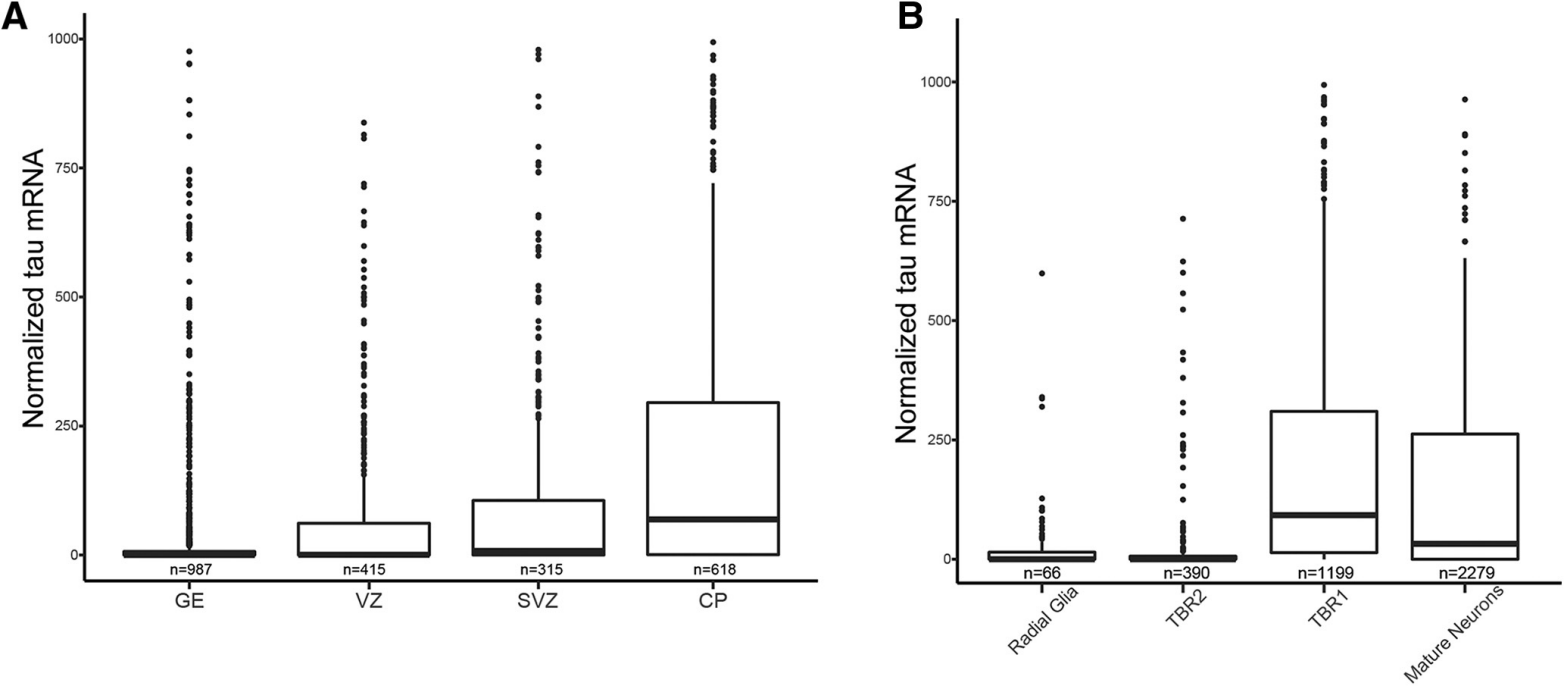
Tau mRNA expression correlates with laminae and neuronal maturation according to sc-RNAseq. ***A***, ***B***, Tau mRNA expression by lamina and neuronal maturation markers in a publicly available scRNAseq dataset. Mature neurons defined as synaptophysin positive and negative for more immature markers, and radial glia as GFAP and nestin positive; 90 values (laminae) and 59 values (cell type) were outside the range and are not shown for clarity. Pseudo-laminae are defined by correlation with microdissected tissue expression patterns in the original publication (Nowakowski et al., 2017).

To better understand the expression pattern of tau mRNA during human fetal brain development, we first analyzed a publicly available scRNA-seq dataset that included 48 cases aged 5.85–37 PCW (Nowakowski et al., 2017). We examined tau mRNA expression as a function of lamina, using ganglionic eminence, VZ, SVZ, and cortical plate pseudo-laminae as defined in the original publication (Nowakowski et al., 2017). Pseudo-laminae were defined in the original publication by correlating gene co-expression networks from sc-RNAseq data with sequencing from microdissected tissue from anatomically defined individual laminae. We found that the SVZ lamina and cortical plate lamina had significantly higher tau mRNA expression than the ganglionic eminence (84.7 [95%CI 33.5, 135.9], p = 0.001 and 257.1 [95%CI 200.9, 313.3], *p* = 2 × 10^−16,^ respectively; Fig. 1*A*). In order to facilitate comparison to RNAscope data, we then defined each cell type according to classic neuronal maturation markers for which RNAscope probes are available. We defined radial glia as *GFAP*^+^/*NES*^+^, intermediate progenitors as *TBR2*^+^, immature neurons as *TBR1*^+^, mature neurons as *SYN*^+^ and negative for more immature markers. Defined this way, immature neurons and mature neurons showed significantly higher tau mRNA expression than radial glia (239.3 [95%CI 135.6, 343.1], *p* = 1.73 × 10^−6^ and 213.5 [95%CI 101.2, 325.7], *p* = 0.0002, respectively; Fig. 1*B*). Intermediate progenitors showed no significant difference when compared with radial glia.

### RNAscope shows co-localization of tau mRNA with TBR1 and SYN in human fetal tissue

**Figure 2.**
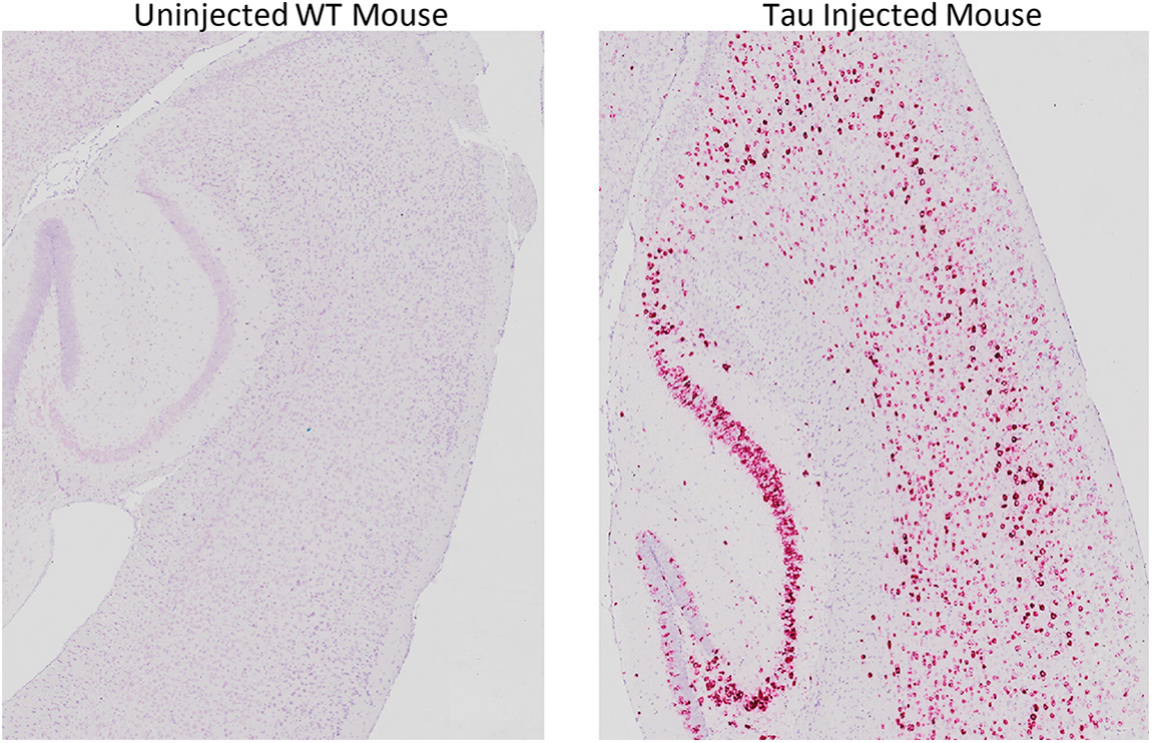
Validation of MAPT RNAscope probe. A FFPE tau P301L AAV-injected mouse brain was stained with a red chromogenic kit (RNAscope 2.5 HD Manual Assay kit) using the same RNAscope MAPT probe used for fluorescent imaging, with a wild-type mouse receiving no injection as a control.

**Figure 3.**
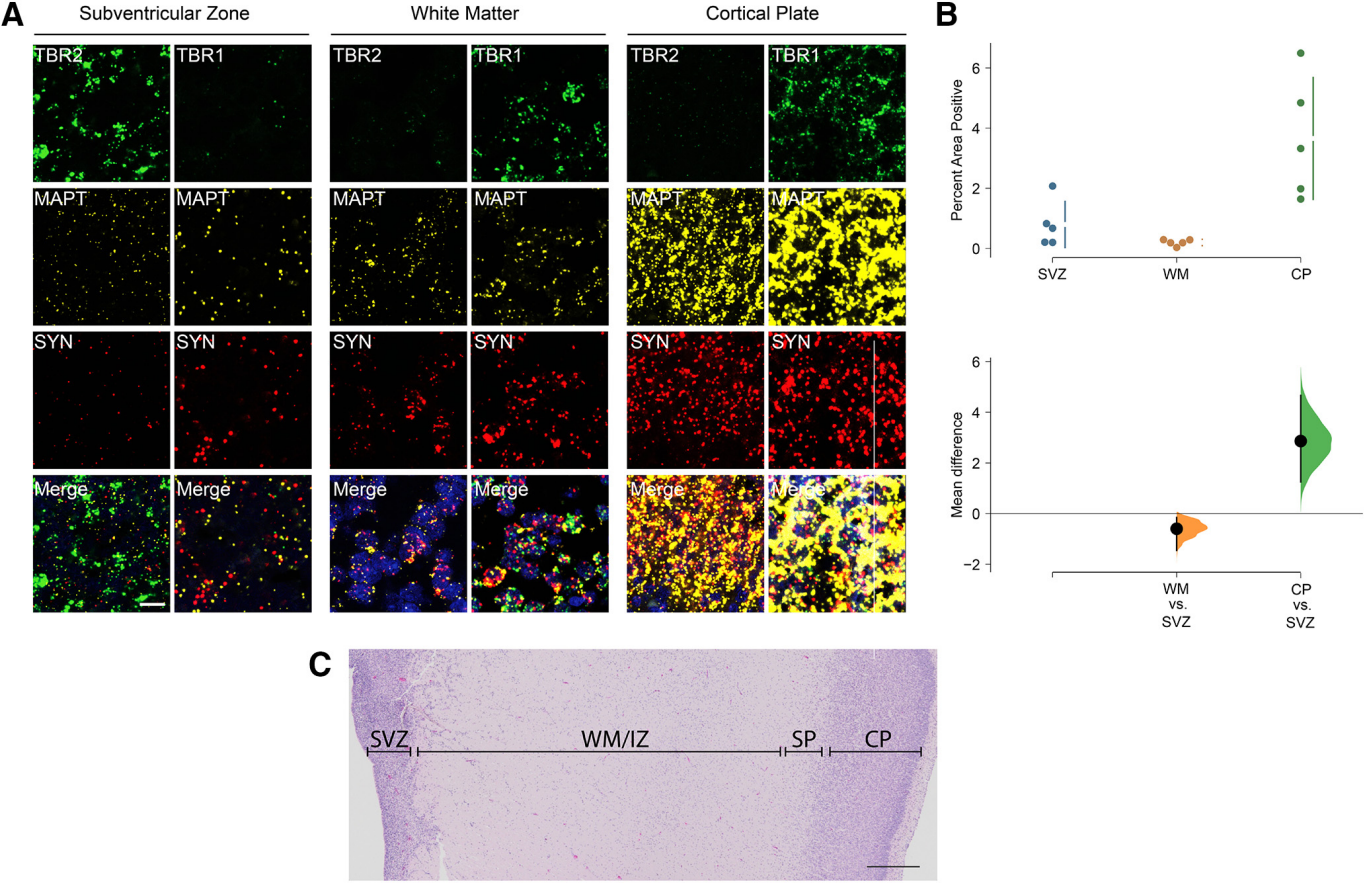
Pattern of tau mRNA expression in the fetal brain by RNAscope. ***A***, Representative MAPT RNA expression by RNAscope in 22 PCW cortical section of fetal brain. All images are from the same representative fetal brain in the designated region (subventricular zone, white matter, and cortical plate). Blue staining represents DAPI. Scale bar: 10 µm. ***B***, The mean difference between samples expressed in a Cumming estimation plot. Raw data are plotted on the upper graph, while the lower graph shows the mean differences as bootstrap sampling distribution. Each mean difference is depicted as a dot, and the confidence interval is indicated by the vertical error bars. Two images of each of the three regions outlined above were taken from five separate cases for quantification. ***C***, Representative H&E-stained image of a FFPE 22 PCW fetal brain tissue section with the areas of interest indicated. Scale bar: 500 μm. CP, cortical plate; SP, subcortical plate; WM, white matter; IZ, intermediate zone; SVZ, subventricular zone.

To validate our human *MAPT* RNAscope probe, FFPE cortical tissue from a tau P301L AAV-injected mouse and wild-type mouse was used with the chromogenic RNAscope kit, which confirmed that only human tau mRNA was labeled (Fig. 2). Once validated, we then examined tau mRNA expression and neuronal maturation marker expression in five human fetal cases ranging from 14 to 24 PCW. The individual cases and causes of death are summarized in Table 1. As expected, in all fetal cases, neurogenic regions had lower but still detectable levels of tau mRNA, while the cortical plate showed the highest levels of expression (Fig. 3*A*). Radial glia (*NES*^+^) and intermediate progenitors (*TBR2*^+^) showed low levels of tau mRNA expression. Both immature (*TBR1*^+^) and mature (*SYN*^+^) neurons showed high levels of tau mRNA beginning in the white matter, with increasing expression levels as they move through to the cortical plate. Additionally, quantification of tau mRNA in all five cases showed an unpaired mean difference between the SVZ and WM of −0.6 [95%CI −1.5, −0.2], *p* = 0.069 and between the SVZ and CP of 2.9 [95%CI 1.3, 4.7], *p* = 0.017 (Fig. 3*B*).

**Table 1:** Summary of case gestational ages and causes of death

PCW	Cause of death	Experiment
14	Idiopathic spontaneous miscarriage	RNAscope
14	Idiopathic spontaneous miscarriage	RNAscope, IHC
18	Idiopathic spontaneous miscarriage	WB
19	Chorioamnionitis	IHC
19	Idiopathic spontaneous miscarriage	WB
19	Idiopathic spontaneous miscarriage	WB
20	Cervical incompetence	RNAscope, IHC
21	Chorioamnionitis	RNAscope
22	Cervical incompetence	RNAscope
22	Chorioamnionitis	IHC
24	Chorioamnionitis	IHC

WB, Western blotting; PCW, post conceptual weeks.

### Tau mRNA expression increases with neuronal maturation using sc-RNAseq from human cortical organoids

**Figure 4.**
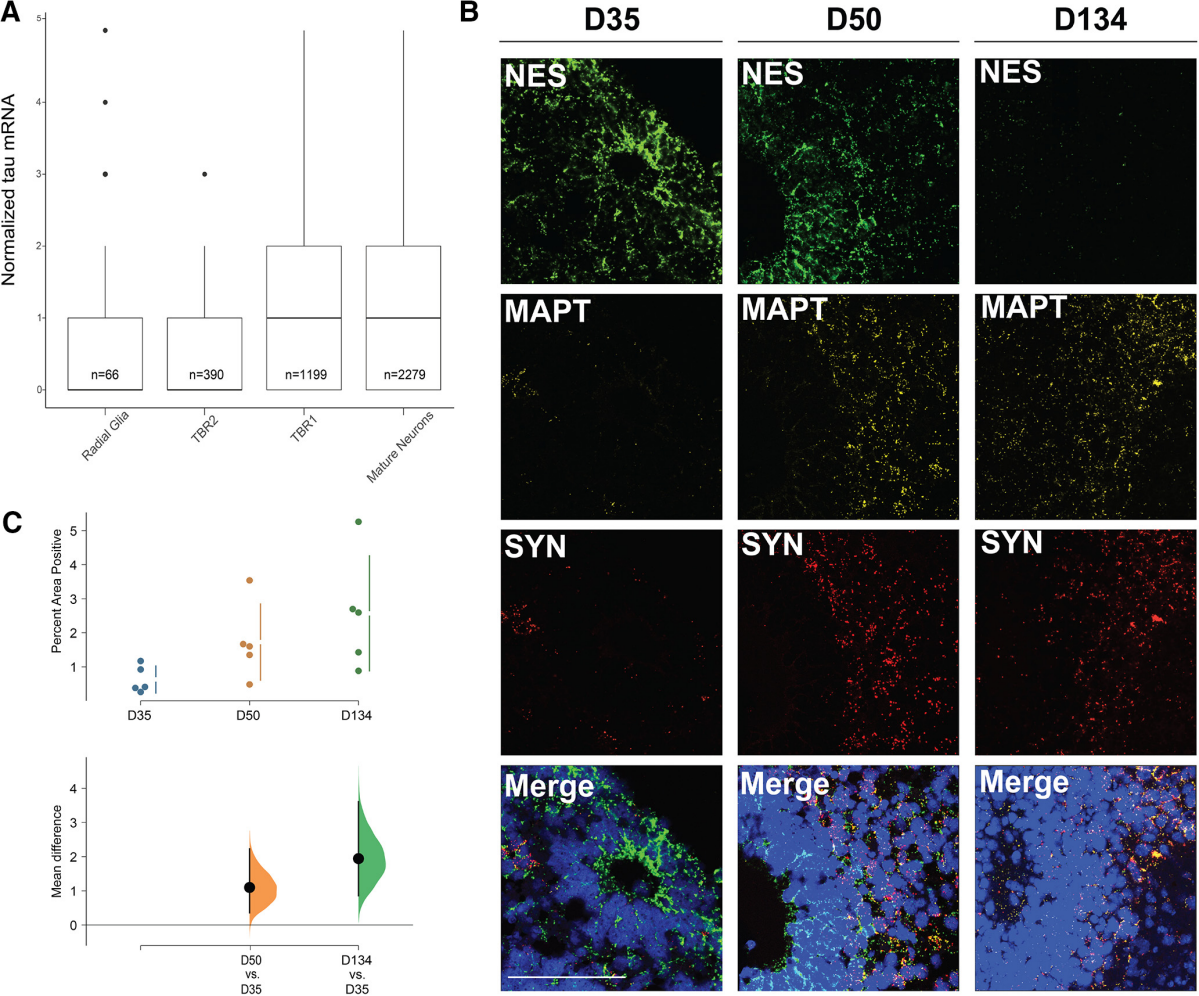
sc-RNAseq and RNAscope of cortical organoids shows tau mRNA expression pattern similar to fetal brain. ***A***, Boxplot of tau mRNA expression in cortical organoids at 105 d in culture. Cell types defined as in Figure 1; 193 points are outside the range and are not shown for clarity. ***B***, Representative MAPT RNA expression by RNAscope in D35, D50, and D134 cortical organoids showing younger organoids with higher *NES*^+^ staining do not express high levels of *MAPT* in their SVZ-like regions, while the older organoids show higher *MAPT*^+^ and *SYN*^+^ co-localization in more mature regions. Scale bar: 100 µm. ***C***, Cumming estimation plots showing mean difference between samples as in Figure 3. Two images of five organoids for each age were used for quantification, as described in the methods section. D, days in culture.

In order to establish iPSC-derived cortical organoids as a model system for tau expression in human fetal cortex, we first analyzed a publicly available scRNA-seq dataset containing three iPSC-derived cortical organoid cultures from three different donors (Yoon et al., 2019). As above, we defined cell types according to neuronal maturation markers that are available for RNAscope, including radial glia (*NES*^+^), intermediate progenitors (*TBR2*^+^), immature neurons (*TBR1*^+^), and mature neurons (*SYN*^+^). The cultured cortical organoids showed significantly greater tau mRNA expression in mature neurons relative to radial glia (respectively, 0.9 [95%CI 0.4, 1.4], *p* < 0.001; Fig. 4*A*). As above, intermediate progenitors showed no significant difference compared with radial glia.

### RNAscope shows co-localization of tau mRNA and neuronal maturation marker SYN in human cortical organoids

To examine tau mRNA expression within iPSC-derived cortical organoids during in vitro differentiation and maturation, we used RNAscope similarly to human fetal tissue. Defining *NES*^+^ as a marker of radial glia and *SYN*^+^ as a marker for mature neurons, we found that *NES*^+^ VZ-like areas have minimal tau mRNA expression by comparison with *SYN*^+^ cortical plate-like areas with high tau mRNA expression (Fig. 4*B*). To assess the developmental progression of tau mRNA expression in human cortical organoids, we quantified the overall expression of tau mRNA in sections of human cortical organoids at D35, D50, and D134 of in vitro differentiation. We identified a significant increase in overall expression of tau mRNA during the in vitro differentiation. Overall, quantification of tau mRNA expression showed that the unpaired mean difference between day in culture D35 and D50 was 1.1 [95%CI 0.4, 2.2], *p* = 0.033; and between D35 and D134 was 1.9 [95%CI 0.9, 3.6], *p* = 0.02 (Fig. 4*C*).

### Tau protein is expressed at low levels in SVZ and germinal matrix in the fetal brain

**Figure 5.**
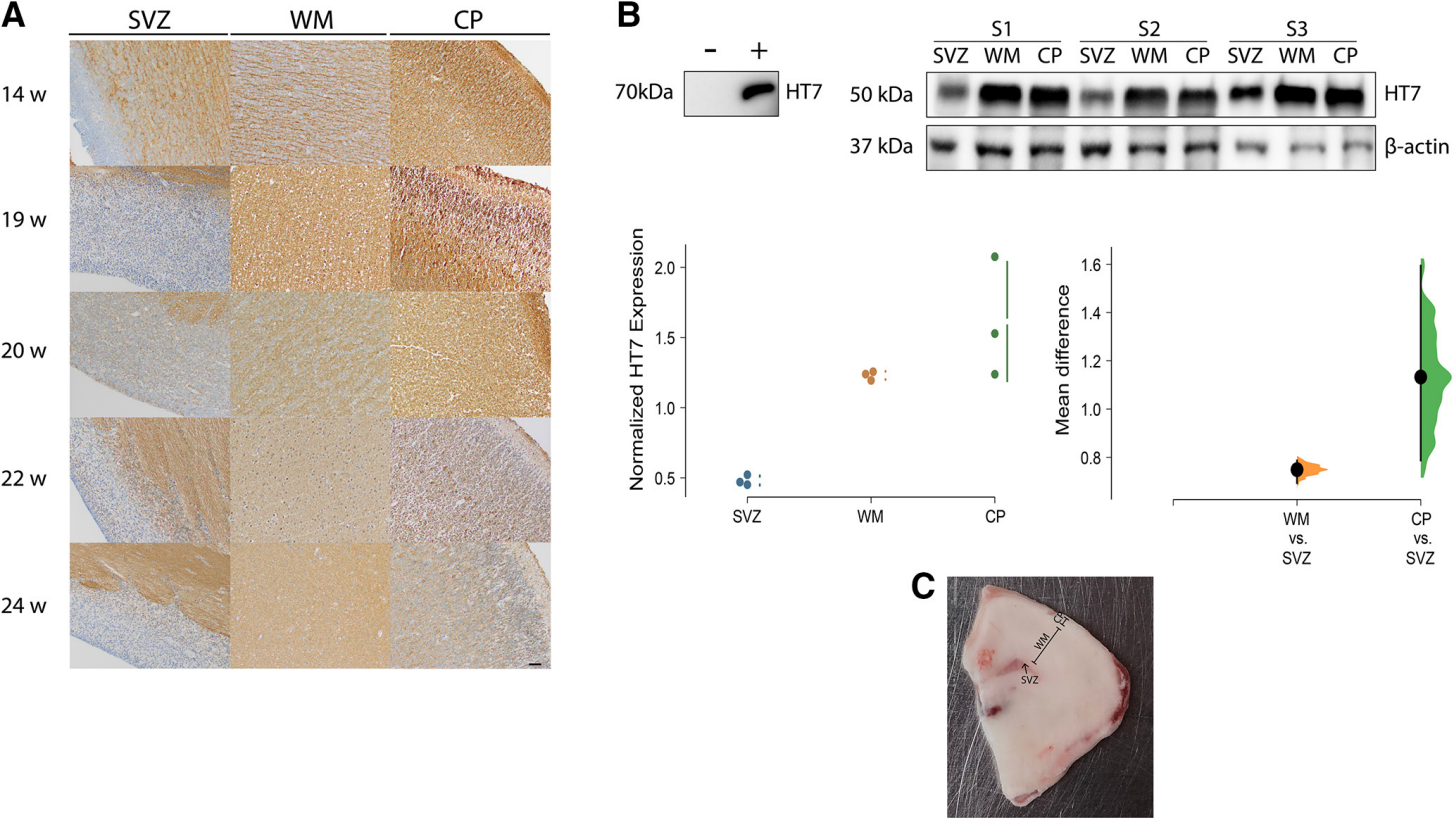
Protein expression is decreased in the SVZ. ***A***, IHC for total tau (HT7 antibody) in FFPE human brain tissue sections. ***B***, Western blotting with HT7 on frozen tissue from corresponding regions with normalized HT7 expression on a Cumming estimation plot showing mean difference between regions as in Figure 3. ***C***, A representative frozen section of brain from a 19 PCW fetus showing the organization of the laminae. SVZ, subventricular zone; WM, white matter; CP, cortical plate. Black bar: 50 µm.

Because we observed low amounts of tau mRNA even in very early neuronal precursors by scRNA-seq and RNAscope, we next assessed protein expression in these same areas by IHC in 5-second trimester fetal cases ranging from 14 to 24 PCW (Table 1). We found that both cortical plate and white matter were diffusely positive but saw only low levels of staining in the germinal matrix or the SVZ (Fig. 5*A*). Similarly, we found decreased tau expression in the germinal matrix and SVZ compared with the white matter and cortical plate by Western blotting (0.7 [95%CI 0.7, 0.8], *p* < 0.001 and 1.1 [95%CI 0.8, 1.6], *p* < 0.001, respectively; Fig. 5*B*).

## Discussion

Based on our data, expression of tau appears to begin very early in development, with tau mRNA detectable in intermediate neuronal precursors and even radial glia. Based on the IHC data, the onset of RNA expression may occur earlier than expression at the protein level. The pattern of tau mRNA expression in cortical organoids derived from hiPSCs appears to be similar, thus validating these as models for studying the development of tau expression. Since human tau shows developmental differences in both splicing and phosphorylation from rodents, the availability of in vitro surrogates for human fetal brain is critically important for progress in this area.

The current findings are consistent with previous work showing that immature regions of the fetal brain, such as germinal matrix, show significantly lower levels of tau mRNA expression compared with cortical plate (Hefti et al., 2018). Others have shown that cortical organoids serve as a high-fidelity model of human fetal brain development (Birey et al., 2017; Blair et al., 2018; Pasca et al., 2019), and our findings extend this by validating organoids as a model of developmental tau expression in humans.

Our study has the advantage of using exclusively human postmortem tissue and cell culture systems (e.g., cortical organoids) and rigorously examining tau expression at both the mRNA and protein levels. It is, however, limited by its observational and retrospective nature. Because of the retrospective nature of our study, our RNAscope experiments were limited to the FFPE sections sampled by the original neuropathologists signing out each case. In addition, although RNAscope is a very robust assay, we found that RNA degradation, presumably due to prolonged postmortem interval, further limits the number of cases suitable for our study. These two factors limit our ability to examine other areas of interest (e.g., hippocampus, basal ganglia). In addition, the publicly available fetal brain scRNAseq data only include cerebral cortex and ganglionic eminence, again excluding these additional areas of interest. Because of the retrospective nature of the study, we are also limited to data available in the autopsy reports, which generally did not include precise postmortem interval. This again limits our ability to account for possible covariates. Although Western blotting revealed a tau protein expression pattern similar to that seen by IHC, our ability to manually dissect SVZ and germinal matrix without including small amounts of white matter and other adjacent structures is limited. This may explain the presence of higher than expected levels of tau protein in immature regions by Western blotting compared with IHC. We did not attempt to quantify individual tau isoforms since it has been previously demonstrated on both the RNA and protein level that fetal tau consists exclusively of the shortest (0N3R) isoform, and the extensive phosphorylation of human fetal tau makes identifying individual isoforms on Western blotting difficult without phosphatase pretreatment (Hefti et al., 2018, 2019).

In conclusion, we have shown that tau mRNA expression increases with neuronal maturation in the developing human brain, and our data suggest that tau mRNA expression is turned on early during neuronal differentiation and may precede the onset of translation. Additionally, we have shown that iPSC-derived cortical organoids serve as a neurodevelopmentally analogous model to the human fetal brain for the purpose of studying the developmental role of the tau protein. Further work should be done using this knowledge to address the impact that tau mRNA and its translational regulators have on protein production in the developing fetal brain.

10.1523/ENEURO.0058-20.2020.supplement Supplementary Statistical Analysis Code. Download Statistical Analysis Code, ZIP file

## Synthesis

Reviewing Editor: Julie Andersen, Buck Institute Decisions are customarily a result of the Reviewing Editor and the peer reviewers coming together and discussing their recommendations until a consensus is reached. When revisions are invited, a fact-based synthesis statement explaining their decision and outlining what is needed to prepare a revision will be listed below. The following reviewer(s) agreed to reveal their identity: NONE.

In this revised manuscript the authors have addressed the majority of points raised in the previous reviews. This includes changing the title to more accurately reflect the content of the manuscript, improvements to the figures and clarification of details within the text and figure legends. The remaining weakness is the lack of functional analyses. However, I agree with the authors that this would involve a substantial amount of additional work. Because all the work has been performed using human fetal tissue in comparison to human iPSC human cortical organoids, the expression data alone does represent a moderate advance in the field. In particular it should help underpin future work aimed at understanding the role(s) of Tau in human cortical development.
